# Fast CU Partition Algorithm for Intra Frame Coding Based on Joint Texture Classification and CNN

**DOI:** 10.3390/s23187923

**Published:** 2023-09-15

**Authors:** Ting Wang, Geng Wei, Huayu Li, ThiOanh Bui, Qian Zeng, Ruliang Wang

**Affiliations:** 1 School of Physics and Electronics, Nanning Normal University, Nanning 530100, China; wangting_nn@163.com (T.W.); pe_lihuayu@126.com (H.L.); oanhbui918@gmail.com (T.B.); zengqian617@foxmail.com (Q.Z.); wrl1201236@yeah.net (R.W.); 2 Technology College of Xiangsihu School, Guangxi Minzu University, Nanning 530100, China

**Keywords:** texture classification, CNN, HEVC/H.265, coding unit partition

## Abstract

High-efficiency video coding (HEVC/H.265) is one of the most widely used video coding standards. HEVC introduces a quad-tree coding unit (CU) partition structure to improve video compression efficiency. The determination of the optimal CU partition is achieved through the brute-force search rate-distortion optimization method, which may result in high encoding complexity and hardware implementation challenges. To address this problem, this paper proposes a method that combines convolutional neural networks (CNN) with joint texture recognition to reduce encoding complexity. First, a classification decision method based on the global and local texture features of the CU is proposed, efficiently dividing the CU into smooth and complex texture regions. Second, for the CUs in smooth texture regions, the partition is determined by terminating early. For the CUs in complex texture regions, a proposed CNN is used for predictive partitioning, thus avoiding the traditional recursive approach. Finally, combined with texture classification, the proposed CNN achieves a good balance between the coding complexity and the coding performance. The experimental results demonstrate that the proposed algorithm reduces computational complexity by 61.23%, while only increasing BD-BR by 1.86% and decreasing BD-PSNR by just 0.09 dB.

## 1. Introduction

As the demand for multimedia content continues to grow, high-definition video services are becoming a sought-after goal [1,2]. To efficiently transmit a huge amount of high-resolution video data, the ITU-T VCEG and ISO/IEC MPEG organizations continuously update and develop video transmission standards. The technique based on CU partition, commonly used in various video coding standards, enhances coding efficiency but at the expense of increased complexity. Take the widely used HEVC/H.265 [3] as an example, although HEVC increases the coding efficiency by nearly 50% over the advanced video coding AVC/H.264 [4], the complex prediction techniques result in a two to four times increase in coding complexity [5]. Among these, the adoption of quad-tree technology in coding unit (CU) partition consumes a significant amount of coding time. It is mainly done by dividing the coding tree unit (CTU) [6] into multiple CUs based on the structure. This process uses a recursive search algorithm that traverses all four CU sizes in the CTU and calculates the rate distribution (RD) costs [7] to determine the best CU partition. Therefore, optimizing the CU partition is an extremely important study to balance complexity and coding performance. Taking into account the most recent generation of video coding standards, such as VVC/H.266 [8], which is 18 times more complex than HEVC [9] and the HEVC is more popular in industrial applications than VVC [10]. The superiority of the algorithm in HEVC is verified without sacrificing generality, thus exploring ways to reduce the complexity of HEVC.

A wide variety of algorithms have been developed to decrease complexity in video coding, primarily by skipping or controlling the traversal rate-distortion optimization (RDO) search at each CU depth. These algorithms can be classified as heuristic or machine learning. Heuristic algorithms [11,12,13] typically extract the prior information based on artificially designed features to assess the complexity of CUs, allowing for the skipping of redundant calculations and optimizing CU partition. Texture information, spatial correlation and statistical RD are commonly used to predict CU depth characteristics. Wang et al. [14] proposed a fast reference image selection strategy that utilizes motion vectors of adjacent coding blocks after performing CU depth pruning based on block texture features and motion information. Liu et al. [15] introduced an adaptive CU selection method that employs depth hierarchy and texture complexity to filter out unnecessary CUs. Moreover, raw redundant pattern candidates can be reduced based on the texture orientation of each prediction unit (PU). Zhang et al. [16] proposed a statistical model-based CU optimization scheme and an intra-prediction model decision. They utilized the texture and depth recursion of spatially adjacent CUs to predict the size range of the current CU. The correlation between the prediction patterns is then utilized to terminate the complete RDO process of each CU depth in advance.

In recent years, machine learning-based algorithms applied to fast CU partitions have gained more and more attention. Among these algorithms, the support vector machine (SVM) based algorithm is a classical approach. Hu et al. [17] proposed a method that utilizes dual SVMs to determine the CU size based on the texture characteristics of the CU. Pakdaman et al. [18] proposed a weight-based adaptive SVM model for deciding the CU partition, thereby reducing the RD cost calculation of traversing all CU sizes. Amna et al. [19] cascaded three SVMs to obtain sufficient features for accurate CU partition. Westland et al. [20] introduced a new algorithm for determining the simplest and most efficient decision tree model, enabling the quick implementation of CU partition. Compared to heuristic algorithms, traditional machine learning methods are able to learn more parameters than heuristic algorithms, making the learned models more accurate. However, although determining the division structure of the CTU by optimization of the decision boundary reduces the computational effort, the global image features are difficult to capture which reduces the accuracy of CU partition.

Recent studies have demonstrated that CNN-based algorithms [21,22,23,24,25,26,27,28,29,30,31] outperform traditional learn-based algorithms in terms of the predicting accuracy for the CU partition because they can automatically extract the features and classify them, and accelerate the speed of CU partition while maintaining high coding performance. Liu et al. [21] first applied CNN to HEVC intra-frame CU partition and deployed it on hardware, thereby reducing encoder complexity. Xu et al. [22] proposed a hierarchical CU partition mapping (HCPM) for the problem of repeated calls to the classifier. Huang et al. [23] combined two state-of-the-art acceleration algorithms, CNN and parsimonious Bayes, based on RD loss to accelerate the classification of CU and PU, respectively. Galpin et al. [24] utilized a CNN-based approach to accelerate the encoding of the intra-frame image slices. Some algorithms design different CNNs to predict the different CU depths. For example, Zhang et al. [25] developed a thresholding model associated with CU depth and quantization parameter (QP) and proposed three different CNNs corresponding to different CU sizes. Zaki et al. [26] proposed a method based on three ResNets to recognize each of the three sizes of CUs. However, the different CNNs deployed at different CU depths make the network more complex and indirectly affect the training speed. To better relate the output of the CNN with the CU partition structure, some algorithms designed the output of the network with a structure corresponding to the CU depth to achieve better prediction. For example, Feng et al. [27] utilized a matrix (8 × 8) to represent the CU depth in a CTU and then predicted each depth value based on a CNN. Tahir et al. [28] used a system based on the integration of online and offline random forest classifiers to segment CUs to accommodate the dynamic nature of video content. Li et al. [29] used a new two-stage strategy for the CU fast partition decision. The CTU partition was predicted in the first stage using a bagged tree model. In the second stage, a CU partition of size 32 × 32 is modeled as a 17-output classification problem. However, although this phased algorithm can effectively improve the coding efficiency, it is extended to the rest of the CU sizes since it is only suitable for the 32 × 32 size CU. Yao et al. [30] constructed a CNN-based dual network structure and applied it to predict CU partition. Li et al. [31] proposed an RL-based CU partition algorithm. The algorithm modeled the decision-making process of CU as a Markov decision process (MDP). Based on the MDP, two different CNNs are used for the depth-independent CU decision learning, thus reducing the computational complexity. This combined structure of two networks, although seemingly convincing, did not yet meet the requirement of making full use of the pixel features of the adjacent CUs.

The above algorithms have studied texture complexity differently and obtained good results. However, there is still potential and need for further exploration of the texture features depending on the different application scenarios. In fact, the high computational complexity incurred during encoding is usually due to dealing with the CUs in the complex texture regions, while the encoding is fast and uncomplicated for the CUs in the smooth texture regions. Therefore, unnecessary division can be reduced by performing partition skipping for the CUs in the smooth texture regions, thus reducing the coding complexity. This paper proposes an algorithm for the fast CU partition based on joint texture classification decisions and machine learning. First, a heuristic-based method is used to determine the texture complexity of the CU which avoids dividing the CUs in the smooth texture regions into smaller sizes. Then, the CUs in the complex texture regions are optimally partitioned using a CNN-based to avoid traversal search. Finally, the whole algorithm process performs three stages of judgment for each CU to ensure that the best CU partition results can be obtained.

In brief, the contributions of this paper can be summarized as follows.

- A classification decision method based on the global and local texture features of the CU is proposed, which divides the CU into smooth and complex texture regions efficiently. Moreover, the CUs in the smooth texture regions will no longer be divided which can avoid the CU redundant partition.
- A novel CNN based on a modified depth-separated convolution is designed for predicting the CU partition in the complex texture regions, thus replacing the RDO process in traditional CU classification and effectively reducing the complexity of the CU partition while maintaining the RD performance.
- Combining the texture classification decision with the proposed CNN achieves both early termination for CUs in smooth texture regions and direct prediction for CUs in complex texture regions using the CNN, thus achieving a good balance between the coding complexity and the coding performance.

## 2. Proposed Fast CU Partitioning Algorithm

In this section, the proposed algorithm for fast CU partition is described in detail. First, the experimental results are observed and the motivation for this paper is presented. Second, the proposed classification decision method for the image texture features is elaborated. Third, the proposed CNN-based is detailed. Finally, a CU partition method based on the texture classification and the CNN is summarized.

### 2.1. Observation and Motivation

**Figure 1:**
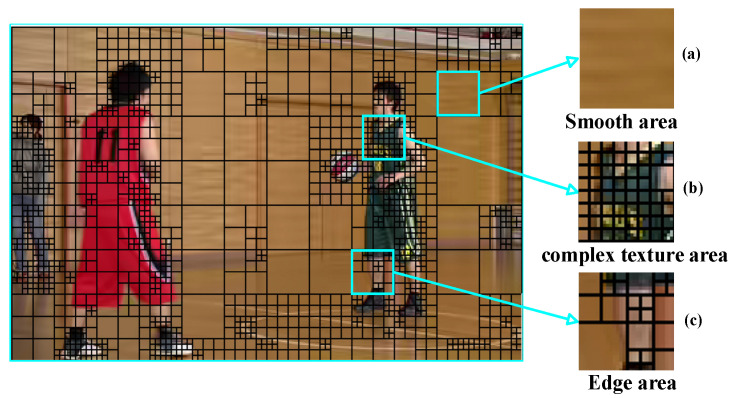
CU partition of Basketball Pass reconstructed frames. (**a**) For smooth area. (**b**) For complex texture area. (**c**) For edge area.

Figure 1 shows the CU partition in the reconstructed video frames (Basketball Pass seq., QP = 32). It can be seen that it tends to have a large CU for the smooth texture region and a smaller CU for the complex texture region in Figure 1a,b, respectively. For the CU of the edge in Figure 1c, it has the above two characteristics. This shows that the large-sized CUs usually appear in smooth texture regions, while the small-sized CUs usually appear in complex texture regions. Therefore, the texture information has a strong correlation with the CU partition.

**Figure 2:**
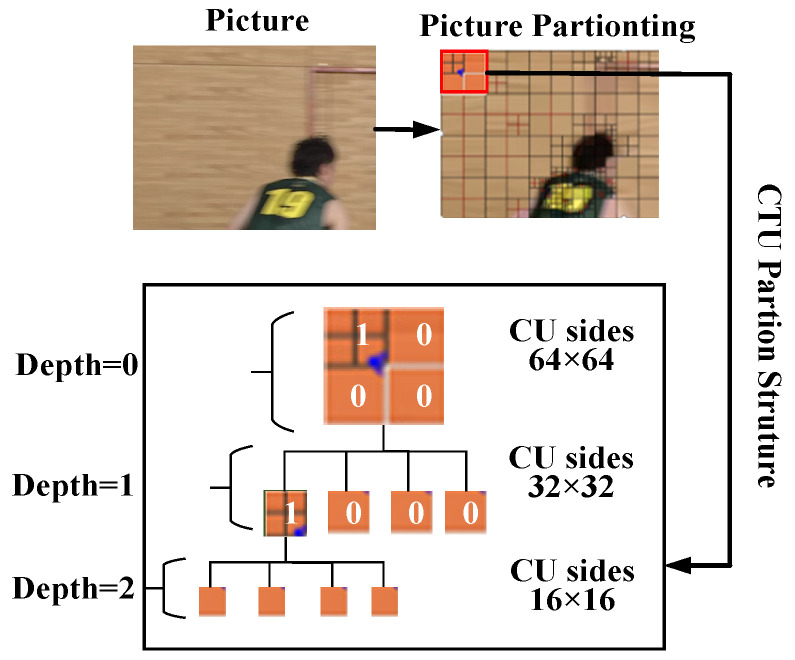
The quad-tree partition structure.

In HEVC, all the CUs of the CTU are traversed based on the quad-tree technique and the RD costs of all CUs are compared for determining the final CU partition. The quad-tree technique is shown in Figure 2, where 0 and 1 mean not splitting and splitting, respectively. The encoding process usually splits the complex texture region into smaller CU after multiple, thus determining the best CU partition structure. In contrast, for the smooth texture regions, only one intra-frame pattern is needed to accurately predict the pixel of the current CTU, and no split into smaller CU is required. This indicates that we can terminate the CU partition of the smooth regions in advance by judging the image texture, which can reduce the coding time by a significant amount.

As mentioned above [11,12,13,14,15,16], the heuristic-based approaches can reduce the complexity of the coding to some extent by skipping the unnecessary CU partition. The machine learning-based method further reduces the encoding complexity by overcoming the shortcomings of the heuristic manual feature extraction. However, the machine learning-based approach does not sufficiently take into account the characteristics of different regions and different CU depths, which limits the degree of complexity reduction. More specifically, the CNN-based CU partition method predicts all CUs identically, regardless of the features of the texture. This is highly unnecessary for smooth regions and may limit the effectiveness of the algorithm. Based on the above observations and analysis, there is an incentive to propose corresponding processing methods for the smooth or complex texture regions, and fully utilize the advantages of heuristic algorithms and machine learning algorithms to reduce the computational complexity while maintaining the coding performance.

### 2.2. Texture Judgment Decision

**Figure 3:**
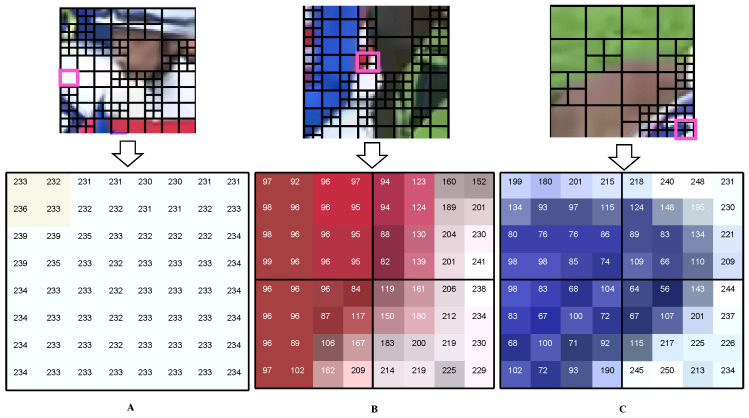
Pixel values in different CU block. (**A**) is the pixel value of the texture flat region, (**B**) is the pixel value of the texture edge region, and (**C**) is the pixel value of the texture complex region.

To measure the CU texture complexity, three different 8 × 8 blocks of pixels from the sequence Race Horses are selected for observation. Considering that the luminance component represents the brightness or intensity information of the image, which is more helpful in perceiving the edge and texture features of the image, the pixel values here are the luminance pixel values, as shown in Figure 3. It can be seen that for block A the pixel values distribution are relatively uniform and the pixel values do not differ significantly. While for areas similar to blocks B and C, the pixel values fluctuate greatly and are not evenly distributed. Therefore, we determine the texture of a CU based on decisions on both global and local metrics.

**Figure 4:**
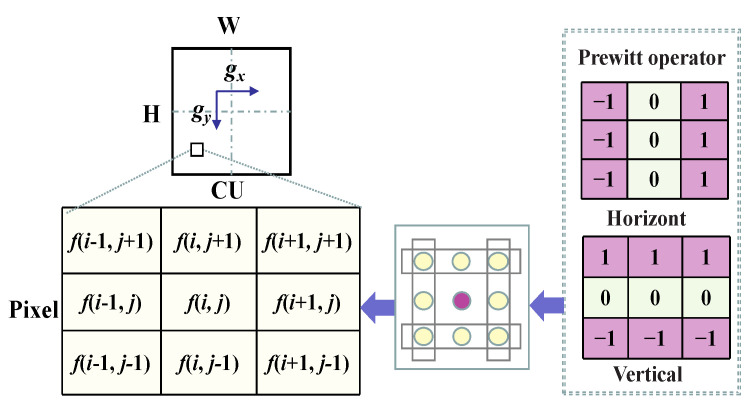
Schematic diagram of CU using the two-directional gradient operator.

The texture information can be obtained by comparing the gradients in different directions. The most common ones are the horizontal and vertical edge information. Prewitt operator [32] can sensitively capture both vertical and horizontal edge information of a pixel. Therefore, these two directional gradient operators are used to calculate the gradient amplitude of the CU and measure the local texture of the CU, as shown in Figure 4. The amplitude of the directional gradient represents the pixel difference between the adjacent pixels along this direction. The closer the difference is to zero, the smoother the region is in this direction. If the gradient difference between the adjacent blocks is large enough, it indicates that they belong to different texture features and need to be divided into smaller CU.

The gradient of CU pixels is calculated as follows:

(1) $$g_{x}=\left|\left[f\left(i+1,j+1\right)+f\left(i+1,j\right)+f\left(i+1,j-1\right)\right]-\left[f\left(i-1,j+1\right)+f\left(j-1,j\right)+f\left(i-1,j-1\right)\right]\right|$$

(2) $$g_{y}=\left|\left[f\left(i-1,j+1\right)+f\left(i,j+1\right)+f\left(i+1,j+1\right)\right]-\left[f\left(i-1,j-1\right)+f\left(j,j-1\right)+f\left(i+1,j-1\right)\right]\right|$$

(3) $$G=\sum_{j=0}^{H-1}\sum_{i=0}^{W-1}\left|g_{x}+g_{y}\right|$$

where *H* and *W* are the height and width of the CU, respectively, whose values are selected from (64, 32, and 16). $g_{x}$ and $g_{y}$ are the horizontal and vertical gradients, respectively. $f\left(i,j\right)$ is the pixel value in the coordinate $\left(i,j\right)$. *G* indicates the gradient amplitude.

For each pixel value $f\left(i,j\right)$ in the CU, the gradient amplitude *G* at its position is computed by $g_{x}$, $g_{y}$. To better characterize the complexity of the current texture, a mean squared root is used to denote the average level of edge strength across the image. First, the average of the gradient amplitudes of all pixel positions in the CU is calculated, and then the root mean square of the gradient amplitude (*GMSR*) is calculated as follows:

(4) $$GMSR=\sqrt{\frac{G}{H\times W}}$$

Since the pixels in the regions with the smooth textures are usually consistent, to reflect the degree of dispersion of pixel values center on the mean. The root mean square error (*RMSE*) is chosen as the global texture complexity metric for determining the CU. When the *RMSE* is larger. It indicates that the CU has a discrete distribution of pixel values, and vice versa, it indicates that the CU has a concentrated distribution of pixel values. The *RMSE* is calculated as follows:

(5) $$Mean=\frac{\sum_{j=0}^{H-1}\sum_{i=0}^{W-1}f\left(i,j\right)}{H\times W}$$

(6) $$RMSE=\frac{\sqrt{\sum_{j=0}^{H-1}\sum_{i=0}^{W-1}\left|f\left(i,j\right)-Mean\right|}}{H\times W}$$

Each CU is evaluated for the global and local complexity. *RMSE* and *GMSR* are used for global judgment and local judgment, respectively.

(7) $$Texture=\left\{\begin{array}{c} Smooth\;texture\;\left(RMSE\leq TH_{A}\;\&\&\;GMSR\leq TH_{B}\right)\\ Complex\;texture\;otherwise \end{array}\right.$$

where $TH_{A}$ and $TH_{B}$ is a predefined threshold. Texture indicates the texture classification of the CU. If the *RMSE* is not greater than the $TH_{A}$, and the *GMSR* is not greater than the $TH_{B}$, the CU is identified as a smooth texture region. Otherwise, it is judged as a complex region.

For CU size larger than 8 × 8, split_flag is used to denote the split flag bit of the current block. If split_flag equals 0, it means no split is needed, i.e., the split is stopped; if it is equal to 1, the split is needed. The expression for the judgment of split_flag is as follows:

(8) $$split\_flag=\left\{\begin{array}{c} 1\;CurCU\to Complex\;texture\\ 0\;CurCU\to Smooth\;texture \end{array}\right.$$

where CurCU denotes the current CU. The CU that is classified as smooth texture can be terminated partition early to avoid more redundant depth partition. Algorithm 1 shows the execution flow of texture classification.
**Algorithm 1:** Texture judgment decision**Input:** Size of current CU, *THA*, *THB*1:*W*, *H* **←** Size of current CU2:R, *ℳ* **←** predefined threshold (*THA*, *THB*)3:calculate *gx*, *gy*, *G*, and *GMSR* by using (1)–(4)4:calculate *Mean* and *RMSE* by using (5) and (6)5:XCOMPRESSCU (*PCurCU)6:    **if** *W*==8 **and** *H*==8 **then**7:        split_flag ← 0   //Terminates the CU recursive partition8:        XCOMPRESSCU (PCurCU)9:    **else if** *RMSE* ≤ R **then**10:        **if** *GMSE* ≤ *ℳ* **then**11:                split_flag ← 012:                XCOMPRESSCU (PCurCU)13:        **else       //**Split the current CU partition14:                XCOMPRESSCU (PCurCUi)15:        **end if   //** The CU recursive partition16:   **else**17:        split_flag ← 118:        PCurCUi ← Pointer to SubCUi19:        XCOMPRESSCU (PCurCUi)20:   **end if**21: **end**

### 2.3. Proposed Network

**Figure 5:**
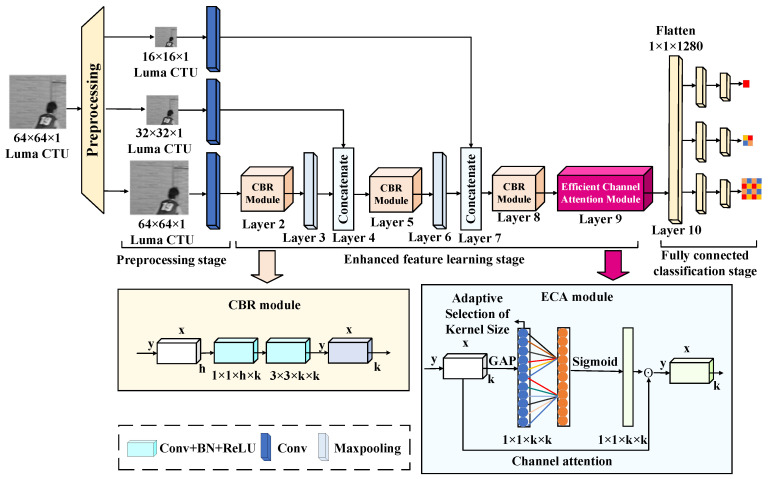
The proposed structure of CNN.

To enable fast prediction of partitioning of CUs with complex textures, Figure 5 shows a novel CNN that enables end-to-end training and learning. The structural depth has 13 layers and can be divided into three stages: the preprocessing stage, the enhanced feature learning stage and the fully connected classification stage. The preprocessing stage enhances the convergence of the features in general. The enhanced feature learning stage leverages the features for low and high-depth feature fusion and end-to-end training for CU partition decisions. The fully connected classification stage fully learns the low-depth and high-depth features to strengthen the global representation of CU partition decision markers. The three stages in the proposed network structure are described in detail below.

Preprocessing stage (Layer 1): Since the luminance component can characterize the visual information better than that of the color component, the proposed CNN mainly extracts the luminance information of video sequences for analysis without loss of generality. A global normalization preprocessing operation is required to ensure that the original luminance component data are taken in the same magnitude range before inputting the data into the network to make the training data fast convergent and accelerate the training speed. To facilitate the feature learning of the different size CUs, the original CTU is down-sampled with three different sizes, respectively. Specifically, the input CU of 64 × 64 size is normalized and down-sampled according to the quad-tree partition size in HEVC and then by means of 4 × 4 convolution layers with non-overlapping features to strengthen the generalization ability of the training CNN. Ultimately, the data of the CU for convolution operations are obtained, in preparation for the later feature extraction stage.

Enhanced feature learning stage (Layer 2–9): To better predict the CU partition, adequate extraction and effective learning of data are required. Inspired by the deeply separable convolution proposed in [33], a similar convolutional structure is used to predict the CU partition. However, unlike [33], in this paper, a novel module named CBR is designed in the newly proposed network. The module consists of two convolutional layers, (i.e., 1 × 1, 3 × 3). Use 1 × 1 convolution to reduce the amount of computation before performing 3 × 3 convolution to reduce the computational effort. In addition, the module combines nonlinear batch normalization (BN) and ReLU during the convolution process to improve the stability and depth of the network, which makes it more suitable for extracting features from the CU partitions.

**Figure 6:**
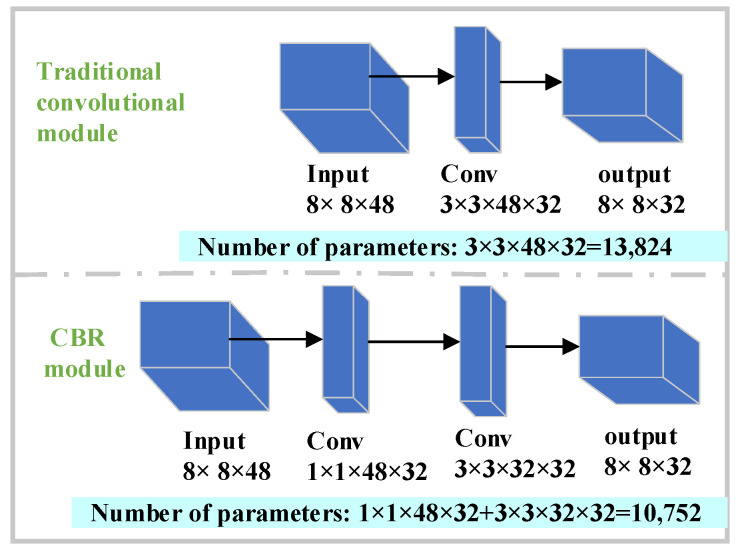
Comparison of the number of parameters between CBR model and traditional convolution.

More specifically, the main structure of the CBR module in this paper is designed as 1 × 1 + 3 × 3 (Conv, BN, and ReLU). First, a 1 × 1 convolution is used to reduce the number of channels as well as realize the channel information interaction, thus avoiding the unlimited increase in the number of channels caused by concatenating the convolution channels in the enhancement feature learning stage. Secondly, the 3 × 3 convolution can reduce the amount of parameter computation compared to other large-size convolutions and enhance the generalization ability and training efficiency of the CNN. It is used after the 1 × 1 convolution to extract the useful features. Furthermore, to explore the computational complexity of the CBR model, Comparing the number of parameters transmitted in the fourth layer of the proposed CNN between the CBR model and the conventional convolutional model is shown in Figure 6. It can clearly be seen that the CBR model has fewer parameters and lower computational costs compared to the traditional convolutional module. This is designed to maintain high performance in extracting the CU features while improving the efficiency and reducing the complexity of the overall model.

After passing the CBR module, the features are fully learned. However, the size of the feature map is not changed, making more redundant information in the deeper features, which indirectly reduces the prediction performance of the network. Therefore, it is necessary to simplify the plane size through the maximum pooling layer to reduce redundant information in the network. The maximum pooling layer kernel size of this network is 2 × 2 and the features do not overlap so that important information can be extracted while avoiding more information loss.

Finally, in order to be able to fully learn the details of CU features of four different sizes, the CU data of the three different down-sampling outputs from the preprocessing stage are added after each maximum pooling layer, thus improving the training efficiency of the network. Note, that due to the different feature map sizes, a separate non-overlapping convolution operation is performed on the down-sampling data to enable concatenation at the end. In addition, a lightweight efficient channel attention (ECA) model [34] is introduced to compensate for the problem of ignoring critical information between network channels in the convolution operation. The ECA model avoids the dimensionality reduction allows for efficient learning of channel information and thus improves accuracy. Without compromising training time, this module is introduced after the output of the convolution operation to ensure performance stability.

Fully connected classification stage (Layer 10–13): The fully connected feature classification is composed mainly of three output and six fully connected layers. The output of the network is designed according to the CU depth. $k_{d}^{\prime }$ denotes the predicted outputs of the fully connected classification stage corresponding to the different depth CU partitions probability with the following expressions:

(9) $$k_{d}^{\prime }=S_{d}\left(CU_{i}\right),\;i=\left\{\begin{array}{c} 64\times 64,\;d=0\\ 32\times 32,\;d=1\\ 16\times 16,\;d=2 \end{array}\right.$$

where *d* corresponds to the CU depth, $S_{d}$ is the classifier corresponding to the different depths and i is the size of CU. The predicted outputs $S_{0}\left(CU_{64\times 64}\right)$, $S_{1}\left(CU_{32\times 32}\right)$, and $S_{2}\left(CU_{16\times 16}\right)$ correspond to the different CU sizes, respectively. First, the input of the fully connected layer needs to convert the data processed by ECA into a one-dimensional vector. Then, the fully connected layer is divided into three branches to correspond to the prediction outputs described above. Finally, each branch passes through two fully connected layers. To correspond to the different depths of CU partition decisions, an HCPM structure [22] is used as the output labels. The network outputs here correspond to 1, 4 and 16, respectively. Note, that the sigmoid function is activated at the output layer to maintain the binary properties of all output labels. To prevent overfitting in the proposed CNN, the loss function is represented by the sum of the cross-entropies to train the network model, which is calculated as follows:

(10) $$L=\frac{1}{N}\sum_{i}L_{i}=\frac{1}{N}\sum_{i}\left(-\left[k_{d_{i}}\times \log\left(k_{d_{i}}^{\prime }\right)+\left(1-k_{d_{i}}\right)\times \log\left(1-k_{d_{i}}^{\prime }\right)\right]\right)$$

where $k_{d_{i}}$ is the truth-ground labels, $k_{d}^{\prime }{}_{i}$ is the predicted output of the CNN and *N* is the total number of samples. The cross-entropy can effectively measure the discrepancy between the model’s predictions and the actual labels, making it a suitable choice for training the network. Table 1 presents the detailed structure and corresponding parameters of the proposed network.

### 2.4. Fast CU Partitioning Algorithm

**Figure 7:**
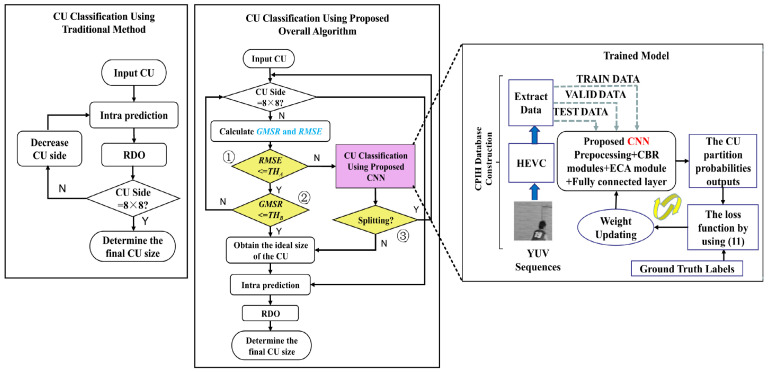
Comparison of the flowchart between the fast and the traditional algorithms. Three layers of metrics corresponding to global judgment *RMSE*, local judgment *GMSE* and prediction judgment of CNN respectively.

An algorithm is proposed based on the CNN combined with CU-based texture discrimination to make full use of texture information. The fast algorithm not only uses the advantages of the heuristic algorithm to skip the unnecessary CU partition in advance but also replaces the complex process of traversal searching RDO in the traditional algorithm based on the advantages of CNN. The general flow of the fast algorithm and the traditional method is shown in Figure 7. Firstly, the collected data is input into the designed network for training. Then, the network is trained cooperatively according to the target loss function. Finally, the trained model is embedded into HEVC, so as to replace the original traversal search process. The proposed algorithm needs to go through three layers of metrics corresponding to global judgment *RMSE*, local judgment *GMSE* and prediction judgment of CNN respectively. Specifically, Before starting to partition the CU, the size of the CU needs to be judged in advance. Except for the 8 × 8 CU, the rest of the CUs first calculate the texture complexity by the metrics *GMSR* and *RMSE*. The global texture complexity of the CU is first measured using *RMSE*. Measures the complexity of the local texture if $RMSE\leq TH_{A}$. If $GMSR\leq TH_{B}$, the CU is considered smooth and no smaller size partition on decision is made. Otherwise, a CNN is used to determine whether it requires further partition. The fast CU partition algorithm can evaluate the current CU texture complexity and select the best CU partition by three layers of metrics.

## 3. Experimental Results and Performance Analysis

### 3.1. Performance Evaluation Index

To evaluate the performance of the proposed algorithm, three evaluation metrics are considered, i.e., the coding time saved evaluates the coding complexity, the BD-PSNR and BD-BR [35] for evaluating the variation in the objective quality of the image. The coding complexity metric is derived from the following equation:

(11) $$\Delta T=\frac{T_{pro}-T_{HM}}{T_{HM}}\times 100\%$$

(12) $$\Delta T_{avg}=\frac{1}{4}\sum_{i=1}^{4}\frac{T_{Pro}\left(QP_{i}\right)-T_{HM}\left(QP_{i}\right)}{T_{HM}\left(QP_{i}\right)}\times 100\%$$

where $T_{HM}$ and $T_{Pro}$ denote the encoding time consumed by the method in the HEVC test platform HM and the proposed algorithm, respectively. The QP is taken from {22, 27, 32, 37} when encoding. The BD-PSNR reflects the residuals between the original and reconstructed pixels, which is calculated as:

(13) $$BD-PSNR=PSNR_{Pro}-PSNR_{HM}$$

where $PSNR_{HM}$ and $PSNR_{pro}$ denote the peak signal-to-noise ratio of the HM and the proposed algorithm, respectively. The BD-BR indicates the average difference in bit rates between the original algorithm and the proposed algorithm as shown as follows:

(14) $$BD-BR=\frac{BD_{rate_{Pro}}-BD_{rate_{HM}}}{BD_{rate_{HM}}}\times 100\%$$

where $BDrate_{HM}$ and $BDrate_{pro}$ denote the output bitrate of the method in the HM and the proposed algorithm, respectively.

### 3.2. Experimental Parameter Configuration

The algorithm in this paper uses the HEVC reference software HM16.5 to evaluate CU partition decisions. The experiments are performed on 18 video sequences from the JCT-VC standard test set [36] and all settings are used as default in CTC. Since only the complexity reduction of the HEVC intra-coding process is considered, the test sequences are encoded with the encoder_intra_main.cfg [37] configuration files. More detailed information on the parameter settings of the test sequence is shown in Table 2. The hardware configuration of the environment is an NVIDIA GeForce RTX 3050 Laptop GPU and AMD Ryzen 7 5800H with Radeon Graphics@3.20 GHz computer, the operating system is Windows 11. The GPU is only there to speed up training and is disabled in testing. The proposed CNN network is implemented on tensorflow [38] version 1.15.4. A robust dataset is necessary for the proposed method to be fully learned. The image dataset CPIH [39] with different resolutions contains CU partition information for 2000 images, which ensures diverse and sufficient data needed to learn to predict CU partitions. Therefore, the proposed CNN is trained and validated using this dataset. The batch size of all training is 64 to predict CU partitions more accurately and the data are trained with one million iterations using a random initialization method during the training process. 

### 3.3. Optimal Threshold Decision

**Figure 8:**
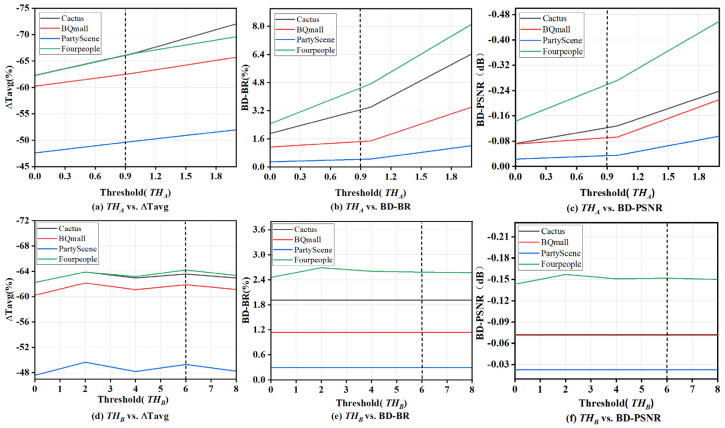
The saved encoding time, BD-BR and BD-PSNR curves varying with the threshold values. Threshold $TH_{A}$ is equal to 0.9 and threshold $TH_{B}$ is equal to 6.

As known in Section 2.4, the threshold directly affects the judgment of texture complexity and subsequent RD performance. To find a suitable threshold that can balance well the computational complexity and RD performance, the threshold experiments are conducted by pre-assignment method. Specifically, to ensure the generality and accuracy of the results, the BQ Mall, Cactus, Party Scene, and Four People sequences with different features and different resolutions are selected for the test. The $\Delta T_{avg}$ curves, BD-BR curves, and BD-PSNR curves varying with the threshold are obtained, as shown in Figure 8. From Figure 8a–c, it can be seen that the $\Delta T_{avg}$ increases steadily with the threshold, the BD-BR and the BD-PSNR change relatively stable when the $TH_{A}$ less than 1 while changing steeply when the $TH_{A}$ is greater than 1. This is due to that as a global threshold, any small changes of $TH_{A}$ will lead to fluctuations in encoding performance and complexity since the features of the entire image are relatively stable. In addition, when the threshold exceeds a certain value, the performance will change significantly. Considering that the threshold $TH_{A}$ greater than 1 may cause a significant change in the bit rate and PSNR, from Figure 8a–c, it can be seen that it is reasonable to choose $TH_{A}$ equal to 0.9 as the threshold. Figure 8d–f shows the $\Delta T_{avg}$ vs. BD-BR and $\Delta T_{avg}$ vs. BD-PSNR curves varying with the threshold $TH_{B}$, respectively, based on the suitable threshold $TH_{A}$ equal to 0.9. It is clear that the fluctuation of the $\Delta T_{avg}$ vs. BD-BR and $\Delta T_{avg}$ vs. BD-PSNR curves with $TH_{B}$ is very small. This is due to that the global metric already takes into account a large number of texture features, leading to a small variation in texture differentiation with local metric thresholds. However, the local metrics can avoid some redundant RD calculations due to their simplicity, thereby reducing time. It can be seen that the encoding time is shortest when $TH_{B}$ equals to 2 and 6, while the changes of BD-BR and BD-PSNR are smoother when $TH_{B}$ equals 6. Therefore, the $TH_{B}$ equal to 6 is empirically chosen as the suitable threshold.

### 3.4. Ablation Experiment

**Figure 9:**
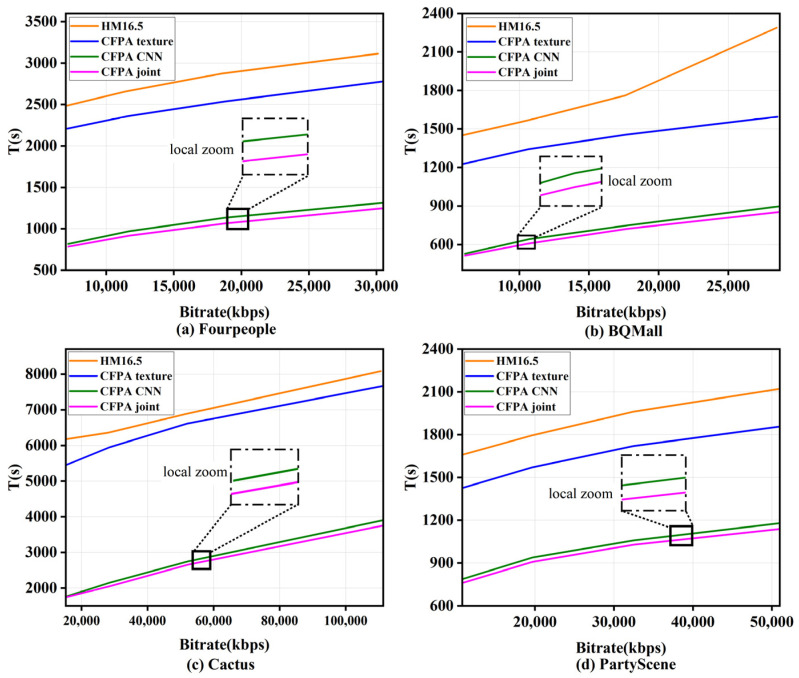
Curves of bitrate vs. encoding time.

**Figure 10:**
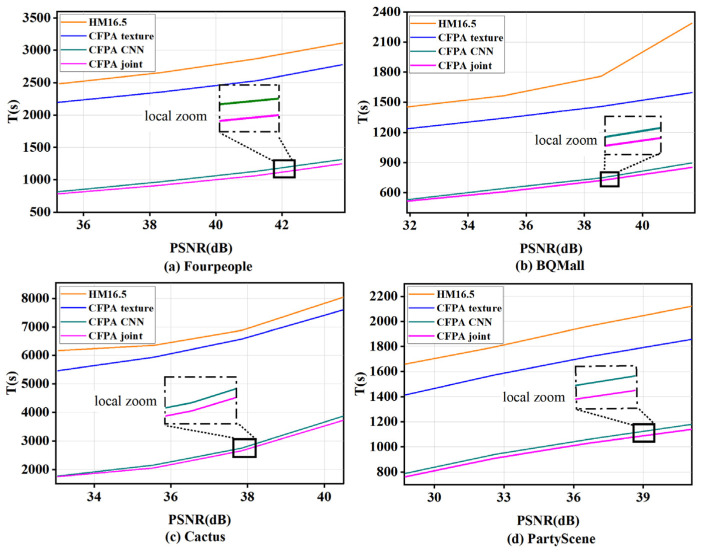
Curves of PSNR vs. encoding time.

For convenience, the proposed texture classification decision, the CNN and the total algorithm are denoted by ${\mathrm{CFPA}}_{\mathrm{texture}}$, ${\mathrm{CFPA}}_{\mathrm{CNN}}$, and ${\mathrm{CFPA}}_{\mathrm{joint}}$, respectively. The original encoder method is denoted as HM16.5. To compare the performance of the above algorithms, this section selected test sequences with different resolutions such as BQ Mall, Cactus, Party Scene, and Four People for encoding testing with QP values {22, 27, 32, 37}, respectively. Figure 9 and Figure 10 show the variation curves of bit rate, PSNR and coding time, respectively. It is confirmed that the ${\mathrm{CFPA}}_{\mathrm{texture}}$, ${\mathrm{CFPA}}_{\mathrm{CNN}}$, and ${\mathrm{CFPA}}_{\mathrm{joint}}$ algorithms can significantly reduce the coding time compared to the original algorithm. In Figure 9 and Figure 10, localized enlarged views of ${\mathrm{CFPA}}_{\mathrm{CNN}}$ and ${\mathrm{CFPA}}_{\mathrm{joint}}$ are shown. Obviously, the proposed ${\mathrm{CFPA}}_{\mathrm{joint}}$ has the least coding time. This advantage is attributed to that the algorithm can avoid the CU partition of the smooth regions and further use the proposed CNN method to predict CU partition of the complex texture regions, consequently effectively reducing many redundant calculations and reducing the coding complexity.

### 3.5. Reduced Complexity and RD Performance Evaluation

Table 3 shows the comparison results of the ΔT (%), BD-PSNR and BD-BR between the ${\mathrm{CFPA}}_{\mathrm{joint}}$ algorithm and the one in HM16.5 by using QP {22, 27, 32, 37} on the standard test sequence. The ${\mathrm{CFPA}}_{\mathrm{joint}}$ algorithm saves −55.76%, −59.92%, −62.75%, and −66.53% of the coding time compared to the original algorithm in the encoder on the four QP, respectively, with only 1.86% increase in BD-BR and −0.090 reduction in BD-PSNR. It is worth mentioning that ${\mathrm{CFPA}}_{\mathrm{joint}}$ can reduce the computational complexity for high-resolution classes (2560 × 1600, 1920 × 1080) by almost 68% and 74%. In particular, for sequences with more complex textures and more intense motion, such as Basketball Drive and Basketball Drill, the ${\mathrm{CFPA}}_{\mathrm{joint}}$ algorithm can reduce the computational complexity by nearly 79% and 76%, respectively. Additionally, for sequences with more smooth textures, such as Kimono, the ${\mathrm{CFPA}}_{\mathrm{joint}}$ can even reduce the computational complexity by almost around 83%. The low complexity of the proposed ${\mathrm{CFPA}}_{\mathrm{joint}}$ is partly attributed to that this algorithm takes a different approach to smooth and complex regions of the texture, respectively, mainly attributed to that the proposed CNN can be used to directly predict the CU partition of the complex texture regions that need high coding complexity. From Table 3, the BD-BR increase is only 1.86% and the BD-PSNR loss is only 0.09 dB. The results show that the ${\mathrm{CFPA}}_{\mathrm{joint}}$ decreases the coding complexity while remaining the sound RD performance.

**Figure 11:**
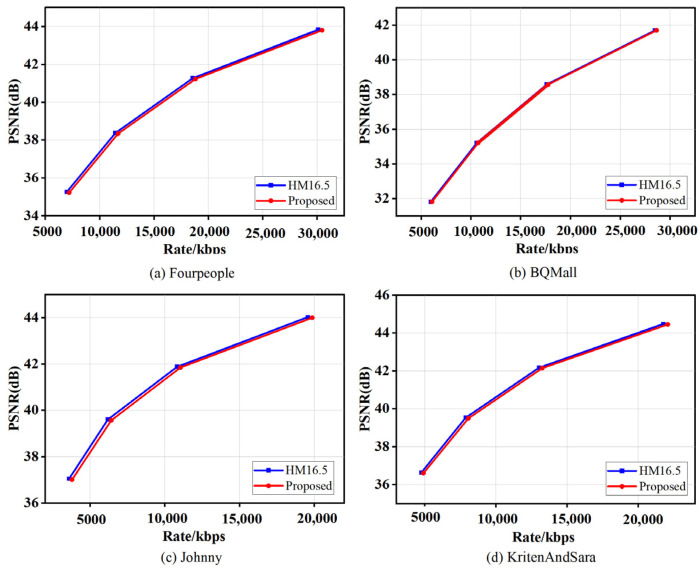
The RD performance of the proposed ${\mathrm{CFPA}}_{\mathrm{joint}}$ and HM16.5 for the four sequences (Basketball Drill, Four People, Johnny, and KritenAndSara).

The four sequences with the large video quality loss (Basketball Drill, Four People, Johnny, and KritenAndSara) are selected for comparison with HM16.5 to assess the RD performance of the ${\mathrm{CFPA}}_{\mathrm{joint}}$ algorithm. Figure 11 shows the RD performance of the proposed algorithm and HM16.5 for the four sequences. It is straightforward to observe that the RD curves of the ${\mathrm{CFPA}}_{\mathrm{joint}}$ and the original algorithm almost coincide. This indicates that the proposed algorithm has good robustness and stability, and even significant video quality loss can be ignored compared to the original encoding.

To further assess the high performance of the algorithm, a comparison was made with three state-of-the-art fast CU partition algorithms implemented in HM16.5, as shown in Table 4. In terms of RD performance, the proposed ${\mathrm{CFPA}}_{\mathrm{joint}}$ algorithm achieved a BD-BR of only 1.86%, which is significantly lower than [21] of 6.19%, [22] of 2.25%, and [23] of 1.90%. Additionally, the BD-PSNR of the ${\mathrm{CFPA}}_{\mathrm{joint}}$ algorithm is −0.09 dB, outperforming [21] of −0.32 dB and [22] of −0.11 dB. Regarding the reduction in encoding time, the ${\mathrm{CFPA}}_{\mathrm{joint}}$ algorithm demonstrated an average complexity reduction of 61.23%, slightly higher than [21] of 61.09%, although slightly lower than [22] of 61.84% and [23] of 61.7%. However, the ${\mathrm{CFPA}}_{\mathrm{joint}}$ algorithm has less loss in BD-BR performance. The main reason is that the proposed CNN can fully utilize the texture features of the CUs with smaller sizes to achieve more excellent judgment.

It is important to note that as encoding time is reduced to a greater extent, there is usually an increase in RD performance loss. Therefore, it is crucial to strike a balance between complexity reduction and RD performance. To further observe the tradeoff between RD performance and coding time savings, a common figure of metric (*FoM*) [40] is used. *FoM* can effectively evaluate the relationship between the two, and the calculation method is as follows:

(15) $$FoM=\frac{BD-BR}{\left|\Delta T_{avg}\right|}\times 100$$

Since *FoM* must strike a good balance between more reduced encoding time and low BD-BR increase, lower *FoM* is desirable [41]. Table 5 displays the *FoM*s for the proposed algorithm and three different state-of-the-art algorithms. It is worth noting that the proposed algorithm has a *FoM* of only 3.0377, which is lower than the other advanced algorithms. Therefore, the algorithm is able to strike a good balance between reducing coding time and RD loss.

**Figure 12:**
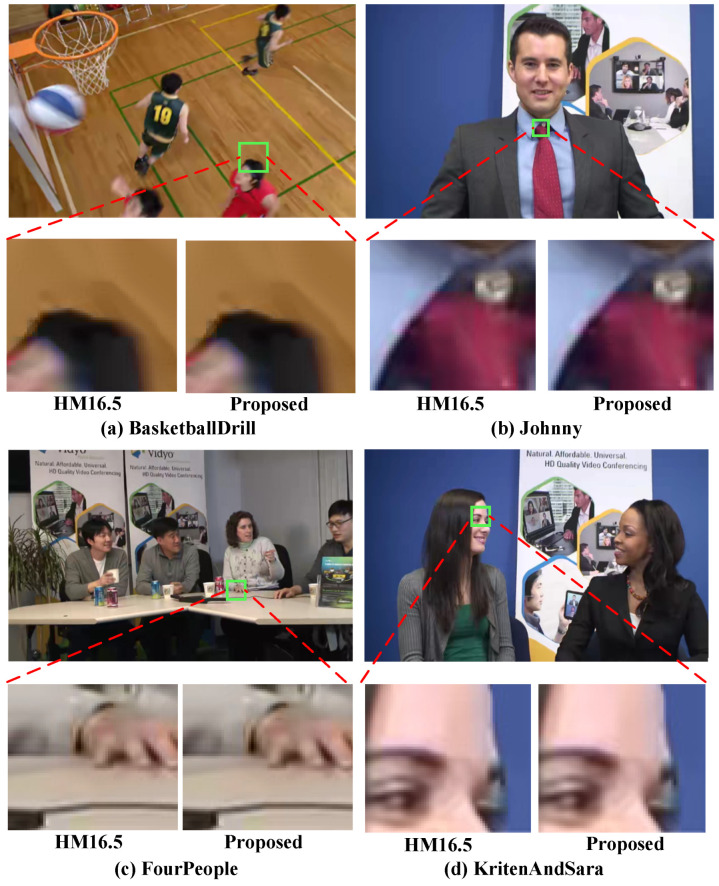
The local zoom regions of the proposed algorithm and HM16.5 for the four sequences (Basketball Drill, Four People, Johnny, and KritenAndSara, QP = 37).

Finally, to assess the subjective quality of the ${\mathrm{CFPA}}_{\mathrm{joint}}$ algorithm, the intra-frames of the four sequences with the large video quality loss (Basketball Drill, Four People, Johnny, and KritenAndSara) are selected for comparison with HM16.5 under QP = 37. Figure 12 shows the local zoom-in regions of four sequences encoded by using the proposed algorithm and the method in HM16.5, respectively. It can be seen that in the local zoom-in regions of the video, the subjective feelings of the two algorithms are almost identical. This indicates that the video quality loss of the proposed algorithm can be ignored.

## 4. Conclusions and Future Work

In this paper, combining the heuristics and the deep learning for predicting the extremely complex CU partition in HEVC is proposed. First, a discriminative method based on image texture can skip the CU partition process of smooth texture regions in advance is proposed. Second, a CNN-based network is designed for CU in complex texture regions for partition prediction, thus improving CU partition efficiency. Finally, image texture recognition decisions are combined with the CNN and embedded in the official HEVC test software HM16.5 to achieve the best CU partition. The experimental results show that the proposed algorithm intra-frame coding complexity by 61.23% on average compared to the HM16.5, while BD-BR is only 1.86%.

In future work, we will consider optimizing the heterogeneous architecture of GPUs to accelerate the processing of CNNs, thus further reducing the encoding time consumption. In addition, we will also focus on adapting our method to better fit the block partitioning structure of VVC and consider applying it to other coding modes. These efforts will further improve the applicability and performance of our approach.

## Figures and Tables

**Table 1:** Detailed parameters of the proposed network.

Input CU Size	64 × 64				
Layer	Layer-1	Layer-2	Layer-3	Layer-4	Layer-5
Output Size	16 × 16 × 16	16 × 16 × 32	8 × 8 × 32	8 × 8 × 48	8 × 8 × 32
Filters	4 × 4, 16	$\left[\begin{array}{c} 1\times 1,\;32\\ 3\times 3,\;32 \end{array}\right]$	$\left[\mathrm{Maxpool},\;2\right]$	Concatenate	$\left[\begin{array}{c} 1\times 1,\;32\\ 3\times 3,\;32 \end{array}\right]$
Layer	Layer-6	Layer-7	Layer-8	Layer-9	Layer-10
Output Size	4 × 4 × 32	4 × 4 × 48	4 × 4 × 80	4 × 4 × 80	1 × 1 × 1280
Filters	$\left[\mathrm{Maxpool},\;2\right]$	Concatenate	$\left[\begin{array}{c} 1\times 1,\;32\\ 3\times 3,\;32 \end{array}\right]$	ECA model	Flatten
Layer	Layer-11	Layer-12	Layer-13	/	/
Output Size	64 128 256	48 96 192	1 4 16	/	/

**Table 2:** Experimental parameter settings of the test sequence.

Class	Sequences	Resolution	Frame Rate (Hz)	Number Frames	Length (s)
A	People On Street	2560 × 1600	30	150	5
	Traffic	2560 × 1600	30	150	5
B	Basketball Drive	1920 × 1080	50	500	10
	BQ Terrace	1920 × 1080	60	600	10
	Cactus	1920 × 1080	50	500	10
	Kimono	1920 × 1080	24	240	10
	Park Scene	1920 × 1080	24	240	10
C	Basketball Drill	832 × 480	50	500	10
	BQ Mall	832 × 480	60	600	10
	Party Scene	832 × 480	50	500	10
	Race Horses	832 × 480	30	300	10
D	Basketball Pass	416 × 240	50	500	10
	Blowing Bubbles	416 × 240	50	500	10
	BQ Square	416 × 240	60	600	10
	Race Horses	416 × 240	30	300	10
E	Four People	1280 × 720	60	600	10
	Johnny	1280 × 720	60	600	10
	KritenAndSara	1280 × 720	60	600	10

**Table 3:** Experimental results of the algorithm on JCT-VC standard test sequence.

Class	Sequence	BD-BR (%)	BD-PSNR (dB)	ΔT (%)			
				QP = 22	QP = 27	QP = 32	QP = 37
A (2560 × 1600)	People On Street	1.95	−0.111	−60.81	−61.70	−61.78	−63.91
	Traffic	2.10	−0.114	−61.26	−65.29	−68.10	−71.53
B (1920 × 1080)	Basketball Drive	4.04	−0.098	−69.18	−74.89	−78.35	−78.95
	BQ Terrace	1.45	−0.088	−52.04	−54.27	−57.98	−61.15
	Cactus	1.91	−0.073	−53.55	−61.40	−66.74	−76.27
	Kimono	1.57	−0.056	−83.44	−83.10	−83.25	−83.96
	Park Scene	1.68	−0.072	−61.03	−72.11	−74.44	−75.97
C (832 × 480)	Basketball Drill	2.72	−0.131	−42.96	−62.12	−62.09	−75.16
	BQ Mall	1.13	−0.071	−62.74	−59.01	−61.12	−64.64
	Party Scene	0.31	−0.023	−46.27	−47.51	−49.36	−54.04
	Race Horses	1.50	−0.095	−48.38	−51.41	−55.90	−61.00
D (416 × 240)	Basketball Pass	2.11	−0.120	−48.21	−52.07	−58.59	−61.96
	Blowing Bubbles	0.67	−0.040	−36.35	−40.03	−45.63	−52.45
	BQ Square	0.21	−0.018	−37.87	−42.62	−44.99	−46.34
	Race Horses	0.92	−0.064	−42.15	−46.78	−50.01	−53.82
E (1280 × 720)	Four People	2.58	−0.151	−59.95	−62.97	−65.49	−68.41
	Johnny	3.90	−0.161	−70.25	−72.18	−73.39	−74.79
	KritenAndSara	2.83	−0.144	−67.18	−69.17	−72.25	−73.27
Average Class A		2.03	−0.113	−61.04	−63.50	−64.94	−67.72
Average Class B		2.13	−0.077	−63.85	−69.15	−72.15	−74.49
Average Class C		1.42	−0.080	−50.47	−55.33	−57.18	−63.84
Average Class D		0.98	−0.061	−41.15	−45.38	−49.81	−53.64
Average Class E		3.10	−0.152	−65.79	−68.11	−70.38	−72.16
Average of Class A–E		1.86	−0.090	−55.76	−59.92	−62.75	−66.53

**Table 4:** Comparison results with the fast CU partition decision making algorithms on sequences.

Class	Sequence	[21]			[22]			[23]		$CFPA_{joint}$		
		BD- BR (%)	BD- PSNR (dB)	$\Delta T_{avg}$ (%)	BD- BR (%)	BD- PSNR (dB)	$\Delta T_{avg}$ (%)	BD- BR (%)	$\Delta T_{avg}$ (%)	BD- BR (%)	BD- PSNR (dB)	$\Delta T_{avg}$ (%)
A	People On Street	3.97	−0.21	−55.59	2.37	−0.13	−61.00	1.89	−61.30	1.95	−0.11	−62.05
	Traffic	4.95	−0.24	−60.84	2.55	−0.13	−70.79	1.74	−63.10	2.10	−0.11	−66.54
B	Basketball Drive	6.02	−0.14	−69.51	4.27	−0.12	−76.32	1.76	−62.90	4.04	−0.10	−75.34
	BQ Terrace	4.82	−0.27	−57.89	1.84	−0.09	−64.72	1.37	−62.30	1.45	−0.09	−56.36
	Cactus	6.02	−0.21	−62.98	2.27	−0.08	−60.96	1.85	−63.90	1.91	−0.07	−64.49
	Kimono	2.38	−0.08	−72.72	2.59	−0.09	−83.53	0.85	−69.00	1.57	−0.06	−83.19
	Park Scene	3.42	−0.14	−66.03	1.96	−0.08	−67.53	1.70	−63.60	1.68	−0.07	−70.89
C	Basketball Drill	12.21	−0.54	−63.58	2.86	−0.13	−52.98	3.48	−63.80	2.72	−0.13	−60.58
	BQ Mall	8.08	−0.47	−52.14	2.09	−0.11	−58.42	2.24	−62.30	1.13	−0.07	−61.88
	Party Scene	9.45	−0.67	−58.75	0.66	−0.04	−44.49	1.70	−56.00	0.30	−0.02	−49.30
	Race Horses	4.42	−0.26	−58.19	1.97	−0.11	−57.12	1.45	−62.40	1.50	−0.10	−54.17
D	Basketball Pass	8.40	−0.46	−64.02	1.84	−0.11	−56.42	2.09	−62.09	2.11	−0.12	−55.21
	Blowing Bubbles	8.33	−0.46	−60.78	0.62	−0.04	−40.54	2.05	−56.00	0.67	−0.04	−43.62
	BQ Square	2.56	−0.21	−46.72	0.91	−0.07	−45.82	1.50	−47.90	0.21	−0.02	−42.95
	Race Horses	4.95	−0.32	−57.29	1.32	−0.08	−55.75	1.65	−57.70	0.91	−0.06	−48.19
E	Four People	8.00	−0.44	−61.54	3.11	−0.17	−71.31	2.30	−62.80	2.58	−0.15	−64.21
	Johnny	7.96	−0.31	−66.55	3.82	−0.15	−70.68	2.61	−69.00	3.90	−0.16	−72.66
	KritenAndSara	5.48	−0.27	−64.72	3.46	−0.17	−74.86	1.88	−64.40	2.83	−0.14	−70.47
Average Class A		4.46	−0.23	−58.22	2.46	−0.13	−65.90	1.82	−62.20	2.05	−0.11	−64.30
Average Class B		4.53	−0.17	−65.83	2.59	−0.09	−70.61	1.51	−64.30	2.13	−0.08	−69.86
Average Class C		8.54	−0.49	−58.17	1.90	−0.10	−53.25	2.22	−61.10	1.41	−0.08	−56.48
Average Class D		6.06	−0.36	−57.20	1.17	−0.08	−49.63	1.82	−56.10	0.98	−0.06	−51.50
Average Class E		7.15	−0.34	−64.27	3.46	−0.16	−72.28	2.26	−65.40	3.10	−0.15	−64.27
Average of Class A–E		6.19	−0.32	−61.09	2.25	−0.11	−61.84	1.90	−61.70	1.86	−0.09	−61.23

**Table 5:** Compared results with the fast CU partition decision algorithm on FoM index.

Algorithm	[21]	[22]	[23]	$CFPA_{joint}$
FoM	10.1326	3.6384	3.0794	3.0377

## Data Availability

The data that supports the findings of this study is available from Wei at this email address, wei_geng@nnnu.edu.cn, upon reasonable request.

## References

[1] Falkowski-Gilski P., Uhl T. (2020). Current trends in consumption of multimedia content using online streaming platforms: A user-centric survey. Comput. Sci. Rev..

[2] Falkowski-Gilski P. (2020). On the consumption of multimedia content using mobile devices: A year to year user case study. Arch. Acoust..

[3] Sullivan G.J., Ohm J.R., Han W.J., Wiegand T. (2012). Overview of the high efficiency video coding (HEVC) standard. IEEE Trans. Circuits Syst. Video Technol..

[4] Wiegand T., Sullivan G.J., Bjontegaard G., Luthra A. (2003). Overview of the H.264/AVC video coding standard. IEEE Trans. Circuits Syst. Video Technol..

[5] Bossen F., Bross B., Suhring K., Flynn D. (2012). HEVC Complexity and Implementation Analysis. IEEE Trans. Circuits Syst. Video Technol..

[6] Guo H., Zhu C., Xu M., Li S. (2020). Inter-Block Dependency-Based CTU Level Rate Control for HEVC. IEEE Trans. Broadcast..

[7] Jamali M., Coulombe S. (2019). Fast HEVC Intra Mode Decision Based on RDO Cost Prediction. IEEE Trans. Broadcast..

[8] Bross B., Wang Y.K., Ye Y., Liu S., Chen J., Sullivan G.J., Ohm J.R. (2021). Overview of the versatile video coding (VVC) standard and its applications. IEEE Trans. Circuits Syst. Video Technol..

[9] Wu S., Shi J., Chen Z. (2022). HG-FCN: Hierarchical Grid Fully Convolutional Network for Fast VVC Intra Coding. IEEE Trans. Circuits Syst. Video Technol..

[10] The Bitmovin Video Developer Report. https://go.bitmovin.com/video-developer-report.

[11] Qi M.B., Chen X.L., Yang Y.F., Jiang J.G., Jin Y.L., Zhang J.J. (2014). Fast coding unit splitting algorithm for high efficiency video coding intra prediction. J. Electron. Inf. Technol..

[12] Zhang W.B., Chen D., Yao X.Y., Feng Y.B. (2018). Fast intra coding unit splitting algorithm based on spatial-temporal correlation in HE-VC. J. Image Graph..

[13] Chen F., Jin D., Peng Z., Jiang G., Yu M., Chen H. (2018). Fast intra coding algorithm for HEVC based on depth range prediction and mode reduction. Multimed. Tools Appl..

[14] Wang X.J., Xue Y.L. Fast HEVC inter prediction algorithm based on spatio-temporal block information. Proceedings of the 2017 IEEE International Symposium on Broadband Multimedia Systems and Broadcasting (BMSB).

[15] Liu X.G., Liu Y.B., Wang P.C., Lai C.F., Chao H.C. (2022). An Adaptive Mode Decision Algorithm Based on Video Texture Characteristics for HEVC Intra Prediction. IEEE Trans. Circuits Syst. Video Technol..

[16] Zhang Y., Li N., Kwong S., Jiang G., Zeng H. (2019). Statistical early termination and early skip models for fast mode decision in hevc intra coding. ACM Trans. Multimed. Comput. Commun. Appl. (TOMM).

[17] Fu B., Zhang Q.Q., Hu J. (2020). Fast prediction mode selection and cu partition for hevc intra coding. IET Image Process..

[18] Pakdaman F., Yu L., Hashemi M.R., Ghanbari M., Gabbouj M. (2021). SVM based approach for complexity control of HEVC intra coding. Signal Process. Image Commun..

[19] Amna M., Imen W., Soulef B., Sayadi F.E. (2022). Machine Learning Based approaches to reduce HEVC intra coding unit partition decision complexity. Multimedi. Tools Appl..

[20] Westland N., Dias A.S., Mrak M. Decision Trees for Complexity Reduction in Video Compression. Proceedings of the 2019 IEEE International Conference on Image Processing (ICIP).

[21] Liu Z., Yu X., Gao Y., Chen S., Ji X., Wang D. (2016). CU partition mode decision for HEVC hardwired intra encoder using convolution neural network. IEEE Trans. Image Process..

[22] Xu M., Li T.Y., Wang Z., Deng X., Yang R., Guan Z. (2018). Reducing complexity of HEVC: A deep learning approach. IEEE Trans. Image Process..

[23] Huang Y., Song L., Xie R., Izquierdo E., Zhang W. (2021). Modeling acceleration properties for flexible intra hevc complexity control. IEEE Trans. Circuits Syst. Video Technol..

[24] Galpin F., Racapé F., Jaiswal S., Bordes P., Léannec F.L., Francois E. CNN-based driving of block partitioning for intra slices encoding. Proceedings of the 2019 Data Compression Conference (DCC).

[25] Zhang Y., Wang G., Tian R., Xu M., Kuo C.J. Texture-Classification Accelerated CNN Scheme for Fast Intra CU Partition in HEVC. Proceedings of the 2019 Data Compression Conference (DCC).

[26] Zaki F., Mohamed A.E., Sayed S.G. (2021). CtuNet: A Deep Learning-based Framework for Fast CTU Partitioning of H265/HEVC Intra-coding. Ain Shams Eng. J..

[27] Feng A., Gao C., Li L., Liu D., Wu F. Cnn-Based Depth Map Prediction for Fast Block Partitioning in HEVC Intra Coding. Proceedings of the 2021 IEEE International Conference on Multimedia and Expo (ICME).

[28] Tahir M., Taj I.A., Assuncao P.A., Muhammad A. (2020). Fast video encoding based on random forests. J. Real-Time Image Process..

[29] Li Y., Li L., Fang Y., Peng H., Ling N. (2022). Bagged Tree and ResNet-Based Joint End-to-End Fast CTU Partition Decision Algorithm for Video Intra Coding. Electronics.

[30] Yao C., Xu C., Liu M. (2022). RDNet: Rate–Distortion-Based Coding Unit Partition Network for Intra-Prediction. Electronics.

[31] Li N., Zhang Y., Zhu L., Luo W., Kwong S. (2019). Reinforcement learning based coding unit early termination algorithm for high efficiency video coding. J. Vis. Commun. Image Represent..

[32] Gao W., Yang L., Zhang X., Zhou B., Ma C. Based on soft-threshold wavelet denoising combining with Prewitt operator edge detection algorithm. Proceedings of the 2010 2nd International Conference on Education Technology and Computer (ICRTC).

[33] Cho S.I., Kang S.J. (2019). Gradient Prior-Aided CNN Denoiser With Separable Convolution-Based Optimization of Feature Dimension. IEEE Trans. Multimed..

[34] Wang Q., Wu B., Zhu P., Li P., Zuo W., Hu Q. ECA-Net: Efficient Channel Attention for Deep Convolutional Neural Networks. Proceedings of the 2020 IEEE/CVF Conference on Computer Vision and Pattern Recognition (CVPR).

[35] Zhang M., Lai D., Liu Z., An C. (2019). A novel adaptive fast partition algorithm based on CU complexity analysis in HEVC. Multimed. Tools Appl..

[36] Bossen F. Common test conditions and software reference configurations. Proceedings of the Joint Collaborative Team on Video Coding (JCT-VC) of ITU-T SG16 WP3 and ISO/IEC JTC1/SC29/WG11, 5th Meeting.

[37] Xu M., Deng X., Li S., Wang Z. (2014). Region-of-Interest Based Conversational HEVC Coding with Hierarchical Perception Model of Face. IEEE J. Sel. Top. Signal Process..

[38] Abadi M., Agarwal A., Barham P., Brevdo E., Chen Z., Citro C., Corrado G.S., Davis A., Dean J., Devin M. (2016). Tensorflow: Large-scale machine learning on heterogeneous distributed systems. arXiv.

[39] Li T., Xu M., Deng X. A Deep Convolutional Neural Network Approach for Complexity Reduction on Intra-mode HEVC. Proceedings of the 2017 IEEE International Conference on Multimedia and Expo (ICME).

[40] Correa G., Assuncao P.A., Agostini L.V., da Silva Cruz L.A. (2015). Fast HEVC encoding decisions using data mining. IEEE Trans. Circuits Syst. Video Technol..

[41] Najafabadi N., Ramezanpour M. (2020). Mass center direction-based decision method for intraprediction in HEVC standard. J. Real-Time Image Process..

